# Primary Cardiac Epithelioid Angiosarcoma in a Latin American Patient: Case Report and Literature Review

**DOI:** 10.1155/2019/2641976

**Published:** 2019-07-30

**Authors:** Dan Morgenstern-Kaplan, Carlos Manuel Aboitiz-Rivera, Ruben Blachman-Braun, María Eugenia Vázquez-Manríquez, Benito Sarabia-Ortega, Mario Enrique Baltazares-Lipp

**Affiliations:** ^1^Centro de Investigación en Ciencias de la Salud (CICSA), Facultad de Ciencias de la Salud, Universidad Anáhuac Mexico Campus Norte, Huixquilucan, Edo. de Mexico, Mexico; ^2^Echocardiography and Hemodynamics Service, Instituto Nacional de Enfermedades Respiratorias, Tlalpan, Mexico City, Mexico; ^3^Department of Pathology, Instituto Nacional de Enfermedades Respiratorias, Tlalpan, Mexico City, Mexico

## Abstract

Cardiac angiosarcoma is a rare and clinically challenging pathology. It is a high-grade primary malignant tumor of the heart tissue that has many variants, of which the epithelioid variant is rarely present in the heart or the great vessels. As with many other cardiac tumors, it is mainly a diagnosis of exclusion and the initial diagnostic test is an echocardiogram followed by a biopsy with immunohistochemistry analysis to ascertain the type of tumor. The differential diagnosis of cardiac tumors is challenging due to the overlapping clinical manifestations with different cardiac tumors and systemic diseases. Cardiac angiosarcomas are often aggressive with a poor prognosis even with treatment. Herein, we present a case of the epithelioid variant of a cardiac angiosarcoma in addition to a thorough review of the recent literature on the clinical manifestation, diagnosis, and treatment of this type of tumors.

## 1. Introduction

Cardiac tumors are rare, and, of these, metastatic tumors to the heart are nearly 30 times more common than primary cardiac tumors. The estimated prevalence for primary cardiac tumors ranges from 0.0017% to 0.33% [1, 2]; of these, 90% are benign, with myxomas being the most frequent. Of the 10% that are malignant tumors, the most common are sarcomas, with undifferentiated pleomorphic sarcoma and angiosarcoma being the most prevalent histological subtypes, accounting for 40% of all the malignant primary cardiac neoplasms [2]. The epithelioid variant is rarely present in cardiac tissue (less than 3% of all angiosarcomas are in the heart and/or great vessels), and it has been reported in many other tissues and organs (i.e., soft tissues and skin, especially in the extremities) [3].

Primary cardiac epithelioid angiosarcoma is extremely rare; to our knowledge, there are only another seven documented reports of this type of tumor [4, 5]. Herein, we present a case of a primary cardiac epithelioid angiosarcoma and a review of the literature.

## 2. Case Report

**Figure 1:**
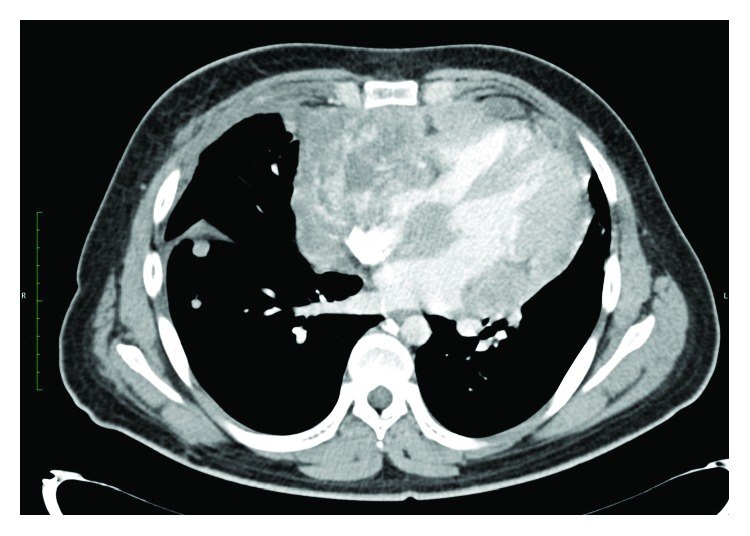
Chest CT scan with IV contrast showing a hyperdense mass within the pericardial space, with infiltration into the myocardium, displacing and compressing adjacent structures.

**Figure 2:**
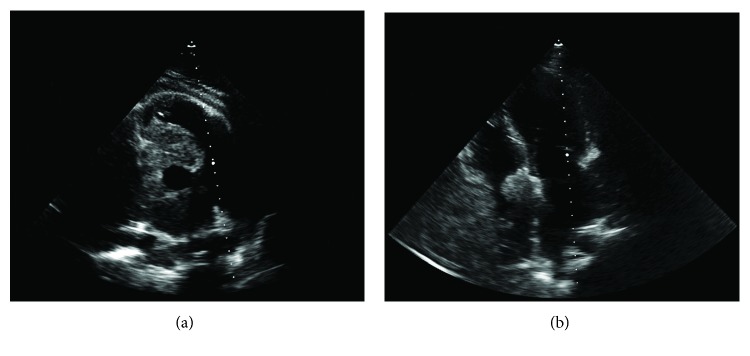
Transthoracic echocardiography. (a) Parasternal short axis view showing tumoral infiltration around the aortic root without obstruction of the outflow tract. (b) Apical 4-chamber view showing myocardial infiltration to the interatrial septum and the free wall of the right atrium and ventricle.

A 20-year-old Hispanic male was referred for cardiologic evaluation with the presence of thoracic oppressive pain and progressively worsening dyspnea, orthopnea, dysphagia, and 8 kg of weight loss in the last month. A chest computerized tomography (CT) scan was performed, reporting the presence of a hyperdense mass within the pericardial space, with infiltration into the myocardium, displacing and compressing adjacent structures, with lymphadenopathy adjacent to the mass. In addition to the mediastinal mass, several hyperdense masses were observed in the right lung (Figure 1). Afterward, transthoracic echocardiography was performed, reporting pericardial effusion with the presence of a mediastinal mass that compromised all four cardiac cavities, infiltrating both atria and partially obstructing the right atrium (Figure 2).

**Figure 3:**
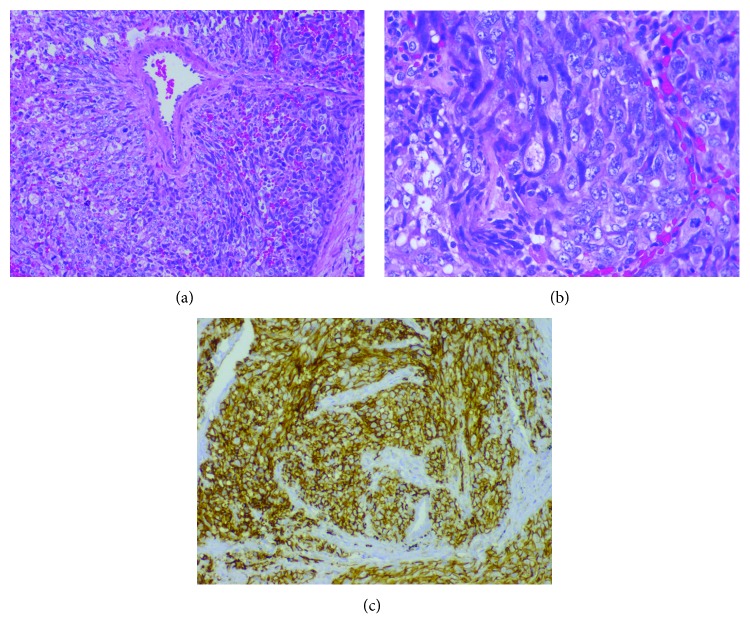
(a) Microphotography H&E (20x). In the center, we can observe a blood vessel surrounded by neoplastic cells in two different patterns: cells forming nests and spindle cells fusing with each other. (b) Microphotography H&E (40x). The tumoral cells are shown in detail, with a medium to large size, scant cytoplasm, oval nuclei of vesicular chromatin, prominent nucleolus, and several mitotic figures. (c) Immunochemistry of the CD31 marker: the neoplastic cells' cytoplasm and membrane are positive, while the bronchial epithelium is negative.

A biopsy was taken (Figure 3), and, macroscopically, the specimen was a 7 × 4 × 2 cm lung parenchyma, with multiple, well-circumscribed solid brown nodular lesions surrounded by collapsed lung tissue. Histological analysis was consistent with a malignant neoplasia of mesenchymal origin with mostly polygonal cells and some spindle-shaped cells arranged around the blood vessels. The cells were pleomorphic with vesiculated nuclei, prominent nucleoli, and a high atypical mitotic rate. The cells were surrounded by a net of capillaries with pleomorphic endothelial cells. In immunohistochemical analysis, neoplastic cells were intensely positive for CD34 and CD31, positive for vimentin, and weakly positive for angiotensin-converting enzyme (ACE), cytokeratin AE1/AE3 (CKAE1/AE3), and epithelial cell adhesion molecule (BerEP4), with a Ki-67 of 50%. This pathological analysis was consistent with the diagnosis of metastatic multifocal epithelioid angiosarcoma.

After the pathological diagnosis was made, the patient was readmitted with symptoms of superior vena cava syndrome. It was then decided to start with the first cycle of chemotherapy with a combination of ifosfamide, epirubicin, and cisplatin. He did not respond to treatment, and the patient with his family decided to forgo treatment. The patient was discharged to palliative care at home.

## 3. Discussion

Cardiac angiosarcomas are rare entities, and the diagnosis and treatment of any cardiac tumor are a challenge for clinicians. Most benign cardiac tumors originate in the atria, more specifically in the left atrium, and cause different clinical presentations depending on the location of the tumor inside the heart, more than its histological subtype. They can cause constitutional and systemic symptoms, obstructive and restrictive cardiac symptoms, arrhythmias, and systemic embolization [1, 6]. The most prevalent symptoms that patients with primary cardiac malignant tumors complain about are exertional dyspnea, chest pain, and palpitations [7]. Though these symptoms are not specific of cardiac malignancies, in the context of systemic symptoms imaging studies of the thorax are recommended to assess the cardiac and thoracic anatomy.

Cardiac tumors are generally diagnosed after excluding other more common pathologies. Echocardiography is the diagnostic tool of choice for the initial evaluation of cardiac neoplasms and the hemodynamic and functional effects of the tumor. The location of the tumor is also of diagnostic use; for example, the most common location for cardiac myxomas is the left atrium, while the most common location for an epithelioid angiosarcoma is the right atrium [8]. Transesophageal echocardiography has 97% sensitivity in detecting cardiac masses, and it can help identify its location, size, shape, attachment, and mobility, characteristics that might provide some insights into the possible mass etiology [9]. Most patients with malignant neoplasms present with disseminated disease at the time of diagnosis, with the lungs being the most frequent site of metastases; however, other metastasis sites include the liver, lymph nodes, bone, adrenal glands, and spleen. Thus, for the staging of this type of malignancies, other required studies include CT scan, magnetic resonance imaging (MRI), echocardiography, and in some cases, positron electron tomography (PET) scan [2]. In the cases of malignant tumors, imaging would show a mass infiltrating into more than one cardiac chamber and into the aorta in most cases; this causes a restriction of heart movement and is highly suggestive of malignancy.

In the case of malignant cardiac tumors, the differential diagnosis includes primarily benign cardiac tumors, which can be suspected clinically with the classic triad of cardiac obstruction, embolism, and constitutional symptoms. Other diagnoses include intracardiac thrombi, vegetations and other infective masses, and calcified lesions. This can be differentiated by imaging studies previously described. Cardiac masses with intramural growth or infiltration often produce conduction defects or arrhythmias that can sometimes be fatal; myocardial infiltration can also produce congestive heart failure secondary to systolic and/or diastolic dysfunction. Other manifestations include myocardial ischemia or infarction secondary to infiltration and compression of coronary arteries. These are the signs that suggest malignancy. Pericardial effusion is reported in both benign and malignant cardiac tumors, but hemorrhagic effusion or tamponade is suggestive of malignancies as well [2].

The lung is a common site of metastasis from soft tissue sarcomas, both from the thorax and the extremities. Leiomyosarcoma (21%), high-grade pleomorphic sarcomas (18%), synovial sarcomas (14%), and liposarcomas (12%) are the most common sarcomas that metastasize to the lung. Furthermore, all these tumors should be in the differential diagnosis of lesions like these in order to determine their origin and should be studied by pathologic analysis with different techniques such as optic microscopy, immunohistochemistry (IHC), or cytogenetics [10].

When the diagnosis is uncertain through imaging studies, biopsy determines the histological type and subtype of the tumor. Microscopic analysis of epithelioid angiosarcomas includes complex interanastomosing vessels lined with epithelioid endothelial cells to solid sheets of atypical epithelioid endothelial cells. Tumor cells are polygonal with prominent nuclear atypia, intracytoplasmic vacuoles, and abundant amphophilic cytoplasm with tumor necrosis often seen [3].

All pathology analyses should include IHC, and a large panel of antibodies must be performed to avoid misdiagnosis because a number of cell markers are shared by many types of tumors, such as pancytokeratins. Epithelioid angiosarcoma is positive for vascular markers such as CD31, CD34, and ERG transcription factor. It is also positive for cytokeratin (indicative of epithelial cells and angiosarcoma), FLI-1, high Ki-67, p53 (positive in 20% of cases), and vimentin (a marker of mesenchymal cells) [3, 11].

IHC is also useful to determine the potential use of targeted therapy in oncology, and in these particular cases it would be useful to search for the presence of important gene markers for current targeted therapy—*KDR* and *FLT-4*—which encode for VEGFR-2 and VEGFR-3, respectively. These markers are receptor tyrosine kinases present in angiosarcomas, and their presence has shown tumors to be sensitive or responsive to sorafenib, a tyrosine kinase inhibitor used in a wide range of cancers [12].

Cardiac angiosarcomas usually have a poor prognosis; they most frequently affect men aged 40 to 50 years, and the prognosis is worse in younger patients, such as the patient in this case report. The mean survival rate of cardiac angiosarcomas is 9.3 ± 4.2 months without surgical treatment; in some cases, where the extent of the disease and the anatomical location allow a complete resection, surgery might be considered a treatment option [13]. On the other hand, chemotherapy with doxorubicin and a combination of antineoplastic drugs has been shown to increase mean survival rate, and radiation therapy is also an option that has been associated with a better survival rate. Pegylated-liposomal doxorubicin has shown promising results in other sarcomas, such as Kaposi's sarcoma, but no randomized controlled clinical trials have been conducted to assess efficacy and safety [14]. Other chemotherapeutic drugs such as cisplatin, cyclophosphamide, dacarbazine, ifosfamide, mitomycin-C, paclitaxel, and vincristine are commonly prescribed, but no standardized treatment algorithms exist as of this moment, although good algorithm proposals have been made [15]. In this patient, palliative care was decided in combination with the medical team, the patient, and his family members. Though information related to the best therapeutic options and outcomes of this type of pathology is insufficient, as just a handful of patients with the diagnosis of primary cardiac epithelioid angiosarcoma has been reported, we hope that this article provides further insight into this pathology.

## 4. Conclusion

Cardiac epithelioid angiosarcomas are rare. Although echocardiography and CT scan might suggest the pretense of a malignant cardiac neoplasm, immunohistochemical diagnosis is required to confirm the diagnosis. When cardiac epithelioid angiosarcomas are diagnosed, prognosis is poor.
